# Photogrammetry as a promising tool to unveil marine caves’ benthic assemblages

**DOI:** 10.1038/s41598-023-34706-7

**Published:** 2023-05-10

**Authors:** Torcuato Pulido Mantas, Camilla Roveta, Barbara Calcinai, Martina Coppari, Cristina Gioia Di Camillo, Veronica Marchesi, Teo Marrocco, Stefania Puce, Carlo Cerrano

**Affiliations:** 1 Dipartimento di Scienze della Vita e dell’Ambiente, Università Politecnica delle Marche, Via Brecce Bianche, 60131 Ancona, Italy; 2 Fano Marine Center, Viale Adriatico 1/N, 61032 Fano, Italy; 3 Stazione Zoologica di Napoli Anton Dohrn, Villa Comunale, 80121 Naples, Italy

**Keywords:** Marine biology, 3-D reconstruction

## Abstract

Traditionally, monitoring approaches to survey marine caves have been constrained by equipment limitations and strict safety protocols. Nowadays, the rise of new approaches opens new possibilities to describe these peculiar ecosystems. The current study aimed to explore the potential of Structure from Motion (SfM) photogrammetry to assess the abundance and spatial distribution of the sessile benthic assemblages inside a semi-submerged marine cave. Additionally, since impacts of recent date mussel *Lithophaga lithophaga* illegal fishing were recorded, a special emphasis was paid to its distribution and densities. The results of SfM were compared with a more “traditional approach”, by simulating photo-quadrats deployments over the produced orthomosaics. A total of 22 sessile taxa were identified, with Porifera representing the dominant taxa within the cave, and *L. lithophaga* presenting a density of 88.3 holes/m^2^. SfM and photo-quadrats obtained comparable results regarding species richness, percentage cover of identified taxa and most of the seascape metrics, while, in terms of taxa density estimations, photo-quadrats highly overestimated their values. SfM resulted in a suitable non-invasive technique to record marine cave assemblages. Seascape indexes proved to be a comprehensive way to describe the spatial pattern of distribution of benthic organisms, establishing a useful baseline to assess future community shifts.

## Introduction

Caves represent a unique pocket of biodiversity in the oceans as on land. The stability of their peculiar environmental conditions can trigger important isolation processes and lead to the evolution and selection of rare endemic species. In the marine environment these conditions, when occurring in the photic zone, offer scientists the opportunity to explore and to study, in accessible “natural laboratories”, the mesophotic and the deep-sea ecosystem biodiversity and functioning^1, 2^, where they can be transposed and studied along a horizontal gradient instead of a vertical one. Moreover, caves can create refuges for various species and reveal unexpected trophic webs^1^. Strong environmental gradients (e.g., hydrodynamics, light, nutrient concentrations, among others) occur towards the inner parts of the caves, shaping their biotic communities and defining different ecological zones^3–6^. And even though the ecological uniqueness and importance of these habitats have been repeatedly acknowledged by the scientific community^7–10^, only a relatively small number of marine caves have been explored and assessed in terms of their biotic component [e.g.,^4, 10–25^].

Global and local stressors strongly affect coastal ecosystems^7, 26^, and marine caves are not an exception. In fact, these habitats are characterised by highly stable and fragile biocenosis^9, 27^, and different studies showed how the synergy of climate change and human activities can deeply modify their communities [e.g.,^7, 28^]. Illegal harvesting (e.g., of *Corallium rubrum* (Linnaeus, 1758), *Lithophaga lithophaga* (Linnaeus, 1758) among others), spearfishing, unregulated visit of divers and tourist boats, and the deep modification of coastal areas, together with the more and more frequent marine heat waves^29^, are also threatening the sessile benthic communities of marine caves, which are known to have a low recovery potential^7, 9, 27, 30^. In the Mediterranean Sea, marine caves are listed as priority habitats by the Habitat Directive (92/43/ECC, code 8330) and included in the Dark Habitats Action Plan^31^. Effective management plans or protection/conservation measures are still lacking^10, 32^. Although some studies have addressed the vulnerability characterising marine caves^7, 9, 33^, it is difficult to generalise the main threats of this habitat at a local scale. For the proper development of targeted and effective measures, *ad-hoc* surveys are crucial to identify the main pressures affecting them^7, 10^.

Traditionally, monitoring approaches to survey marine caves have been constrained by equipment limitations and the strict safety protocols of cave diving^34^. Different methods have been used to describe the biodiversity hosted in these enclosed habitats, including among others, photo-quadrats, linear transects or video surveys^10, 25, 35–38^. Nowadays, the development of new technologies and the minimization of underwater equipment contributed to the emergence of new tools: such as vehicles (e.g., Autonomous Underwater Vehicles (AUVs)), sensors (e.g., compact echosounders), and methods (e.g., Structure from Motion (SfM) photogrammetry), which opened new possibilities to assess changes also occurring inside caves^39–41^. Thanks to its ability to digitally reconstruct a whole scenario from a series of overlapping images, SfM-photogrammetry is considered a cost-effective technique to monitor a great variety of underwater environments from a three-dimensional (3D) perspective^42–56^. Additionally, it offers the possibility to better assess organisms with complex architectures, constituting a suitable method for in-situ non-invasive evaluations^57^. The feasibility of this approach has been confirmed in different habitats, not only by its increased number of applications in the scientific literature, but also by its implementation in citizen science initiatives^44^, as its recent inclusion among citizen science protocols for the monitoring of Marine Protected Areas in the Mediterranean (e.g., protocol 11 of the Interreg MED MPA Engage project)^56^.

In this context, the objective of the current study was to explore the potential of SfM-photogrammetry to assess the abundance and spatial distribution patterns of the sessile benthic assemblages associated with a semi-submerged marine cave, considering, as a case study, a cave in the North Adriatic Sea. We focussed our attention mainly on the sponge diversity, since Porifera represents the predominant taxon within the study cave, thus constituting an essential component in this environment, and more generally acting as ecosystem engineers, driving substrate and nutrient availability^58–60^. Additionally, a special emphasis was paid to *L. lithophaga’*s hole distribution and densities, due to the still current illegal fishing activities occurring in the area (Fig. S1), despite being strictly protected under international directives, agreements, and conventions^27^. The obtained results using photogrammetry were compared with those achieved through a more traditional approach, by simulating photo-quadrats deployments over the produced orthomosaics, to assess their efficiency in capturing biodiversity.

## Results

### Benthic sessile community assessment

**Figure 1:**
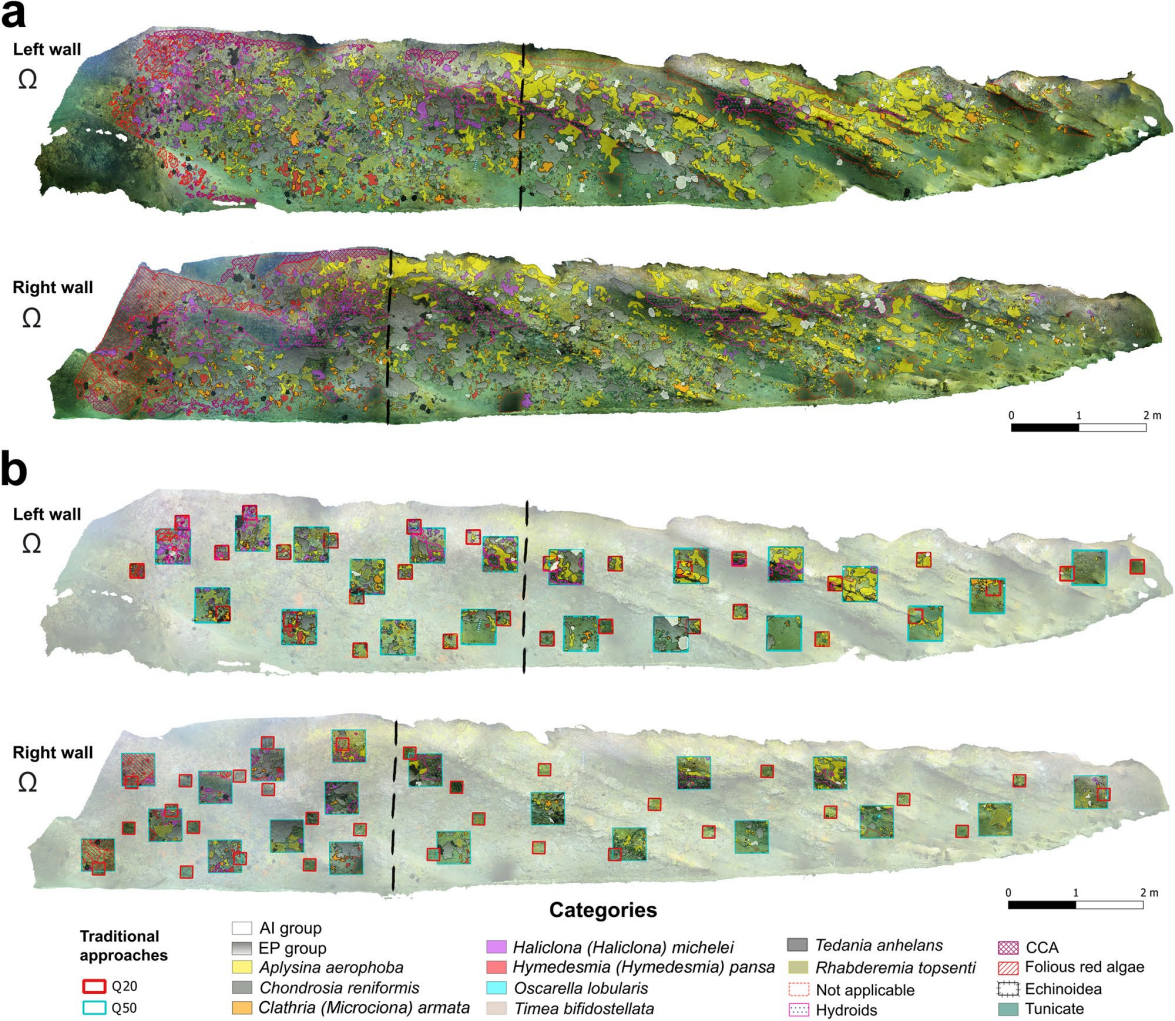
(**a**) Cave walls orthomosaics segmented by the categories defined in Table 2; (**b**) distribution of the randomly deployed quadrat approaches. Dashed lines delimit the semidark and dark areas. Q20 = 20 × 20 cm quadrats; Q50 = 50 × 50 cm quadrats. The symbol Ω indicates the cave entrance. For categories’ acronyms see Table2. Created using QGIS software version 3.12 (http://www.QGIS.org).

Thanks to the photogrammetric approach it was possible to obtain a 3D digital reconstruction for the whole cave (Video S1). Additionally, two orthomosaics were produced from the orthoplane of each of the walls (Fig. 1a), corresponding to a total projected area of 61.2 m^2^ (24.5 m^2^ and 36.7 m^2^ for the semidark and dark zone, respectively) (Table 1). Overall, by coupling photogrammetry and the sponge samples analysis, a total of 22 sessile taxa were identified, including Rhodophyta (2), Porifera (13), Hydrozoa (1), Serpulidae (1), Bilvavia (1) and Tunicata (4) (Table 2). In addition, 2 vagile taxa, the shrimp *Palaemon serratus* and the echinoid *Paracentrotus lividus*, were observed (Table 2). Among all identified taxa, only two (*Aplysina aerophoba* and *P. lividus*) are under protection, being listed in the Annex II and III of the SPA/BD Protocol of Barcelona Convention.

**Table 1:** Sampling protocols applied for the cave community characterization.

Protocol	Sampling units	Sampling area	Description	References
SfM	Whole cave	61.2 m^2^	Reconstruction of the whole cave habitat	Palma et al.^45^, Bayley and Mogg^49^
Q20	60	2.4 m^2^	Random deployment of 15 quadrats per cave zone	Bianchi et al.^36^
Q50	40	10.0 m^2^	Random deployment of 10 quadrats per cave zone	Bianchi et al.^36^

Q20 = 20 × 20 cm quadrats; Q50 = 50 × 50 cm quadrats; SfM = Structure from Motion photogrammetry.

**Table 2:** List of identified species and benthic categories with the corresponding coverage per category, expressed as total cover for Structure from Motion (SfM) photogrammetry and average cover (av) (± standard deviation, sd) for 50 × 50 cm quadrats (Q50) and 20 × 20 cm quadrats (Q20).

Benthic categories	Group composition	SfM (m^2^)			Q50 (area ± sd, m^2^)			Q20 (area ± sd, m^2^)		
		Semidark	Dark	Whole	Semidark	Dark	Whole	Semidark	Dark	Whole
Porifera										
AI group	*Aaptos aaptos* (Schmidt, 1864) *Ircinia variabilis* (Schmidt, 1862)	0.013	0.434	0.447	0.010 ± 0.005	0.012 ± 0.019	0.012 ± 0.019	–	0.027 ± 0.047	0.027 ± 0.047
* Aplysina aerophoba* (Nardo, 1833)*		1.103	5.31	6.413	0.008 ± 0.018	0.020 ± 0.034	0.015 ± 0.028	0.035 ± 0.047	0.09 ± 0.13	0.07 ± 0.11
* Chondrosia reniformis* (Nardo, 1847)		0.031	0.024	0.055	0.0002	–	0.0002	–	–	–
* Clathria (Microciona) armata* (Bowerbank, 1862)		0.606	0.744	1.35	0.004 ± 0.006	0.005 ± 0.008	0.004 ± 0.007	0.018 ± 0.027	0.018 ± 0.031	0.018 ± 0.029
EP group	*Erylus mammilaris* (Schmidt, 1862) *Penares euastrum* (Schmidt, 1868)	2.921	4.008	6.929	0.049 ± 0.079	0.026 ± 0.057	0.037 ± 0.069	0.12 ± 0.11	0.11 ± 0.15	0.11 ± 0.13
* Haliclona (Haliclona) michelei* Van Soest & Hooper, 2020		1.071	0.319	1.39	0.007 ± 0.009	0.004 ± 0.005	0.006 ± 0.008	0.035 ± 0.034	0.03 ± 0.03	0.034 ± 0.033
* Hymedesmia (Hymedesmia) pansa* Bowerbank, 1882		0.324	0.033	0.357	0.008 ± 0.140	0.002 ± 0.050	0.007 ± 0.013	0.023 ± 0.040	–	0.02 ± 0.04
* Oscarella lobularis* (Schmidt, 1862)		0.047	0.005	0.052	0.001 ± 0.001	0.005 ± 0.007	0.002 ± 0.002	0.007 ± 0.008	–	0.007 ± 0.008
* Rhabderemia topsenti* Van Soest & Hooper, 1993		0.836	0.666	1.502	0.023 ± 0.026	0.016 ± 0.042	0.021 ± 0.033	0.070 ± 0.068	0.08 ± 0.13	0.08 ± 0.11
* Tedania anhelans* (Vio in Olivi, 1792)		0.294	0.167	0.461	0.007 ± 0.010	0.011 ± 0.015	0.008 ± 0.012	0.030 ± 0.024	0.04 ± 0.09	0.031 ± 0.061
* Timea bifidostellata* Pulitzer-Finali, 1983		0.483	0.09	0.573	0.006 ± 0.014	0.012 ± 0.042	0.008 ± 0.023	0.029 ± 0.046	–	0.029 ± 0.046
Others										
Crustose Coralline Algae (CCA)		0.757	–	0.757	0.007 ± 0.011	–	0.007 ± 0.011	0.027 ± 0.032	–	0.027 ± 0.032
Folious red algae		1.974	–	1.974	0.06 ± 0.15	–	0.06 ± 0.15	0.25 ± 0.25	–	0.25 ± 0.25
Hydroid assemblages		1.776	1.311	3.087	0.051 ± 0.092	0.09 ± 0.12	0.063 ± 0.11	0.19 ± 0.19	0.13 ± 0.13	0.16 ± 0.17
Tunicates	Didemnidae Giard, 1872 Ascidiidae Herdman, 1882 Styelidae Herdman, 1881 Pyuridae Hartmeyer, 1908	0.153	0.441	0.594	0.003 ± 0.004	0.002 ± 0.002	0.002 ± 0.003	0.011 ± 0.012	0.013 ± 0.012	0.012 ± 0.012
Bare substrata		11.876	21.735	33.611	0.045 ± 0.120	0.61 ± 0.23	0.10 ± 0.22	0.22 ± 0.23	0.57 ± 0.23	0.33 ± 0.28
Not considered for cover calculation										
* Lithophaga lithophaga* (Linnaeus, 1758)*		–	–	–	–	–	–	–	–	–
Serpulids		–	–	–	–	–	–	–	–	–
* Palaemon serratus* (Pennant, 1777)		–	–	–	–	–	–	–	–	–
* Paracentrotus lividus* (Lamarck, 1816)*		–	–	–	–	–	–	–	–	–
Not applicable		0.284	1.44	1.724	–	–	–	–	–	–

* = listed in the Annex II and III of the SPA/BD Protocol of Barcelona Convention.

**Figure 2:**
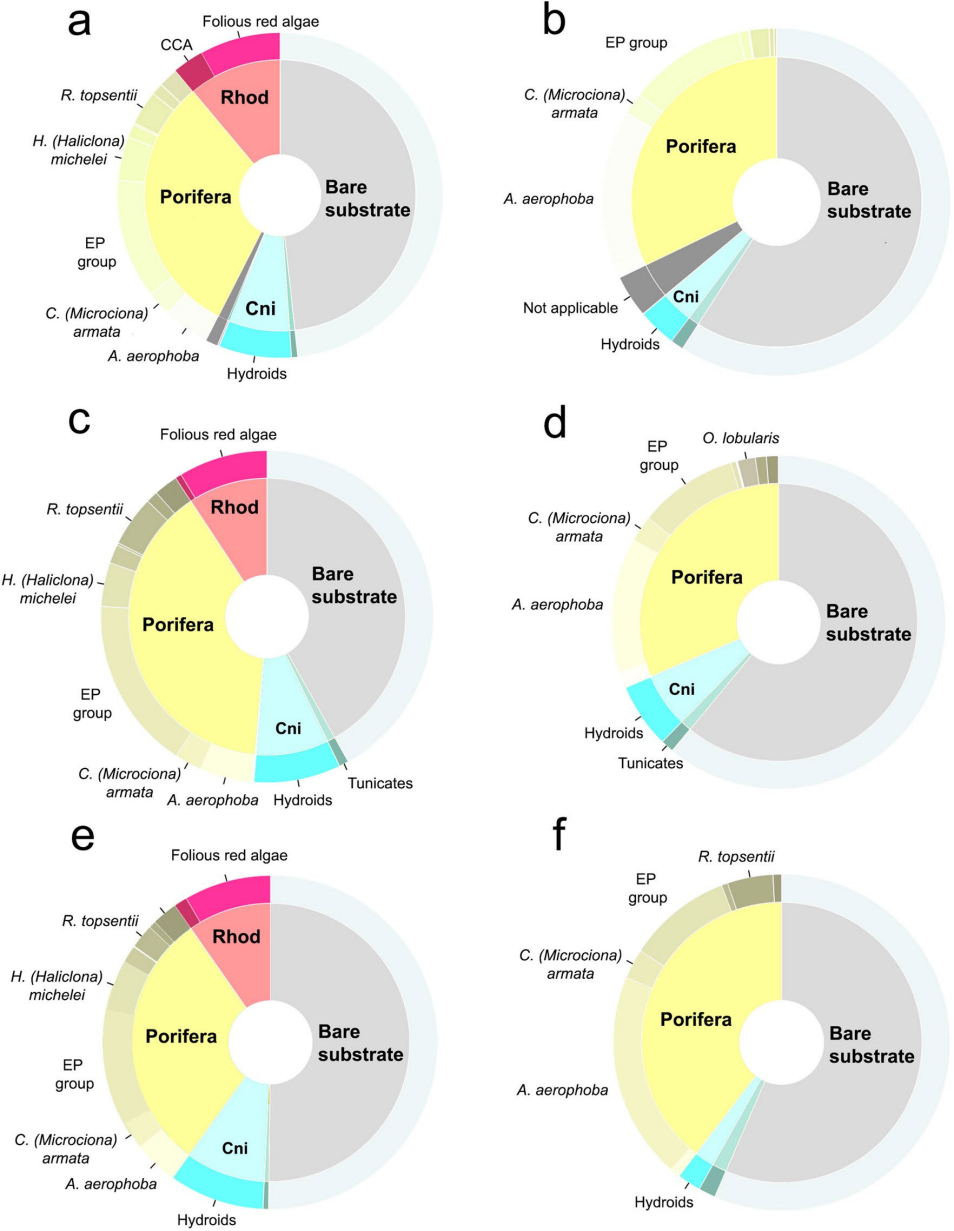
Ring-charts representing the percentage cover of the sessile benthic taxa identified and of the bare substrate per: Structure from Motion Photogrammetry in the (**a**) semidark and (**b**) dark zones; 50 × 50 cm quadrat approach in the (**c**) semidark and (**d**) dark zones; and 20 × 20 cm quadrat approach in the (**e**) semidark and (**f**) dark zones. For categories’ acronyms see Table2.

Since it was not possible to differentiate some of the sponge species by simple imagery, for the analyses, several species were clustered into groups, allowing to identify a total of 16 benthic categories (Table 2). The area covered by each category was extracted separately for each eco-zone (Table 2; Figs. 1a and 2a). Apart from the Rhodophyta (crustose coralline algae (CCA) and folious red algae), all the other categories were shared between the two biocoenosis, with a gradual decrease in the biotic cover towards the inside part of the cave, where the percent coverage of bare substrate reached the 55% (Figs. 1a and 2a). In both cave biocoenoses, Porifera resulted as the prevalent phylum, with a total of 31.4% and 32.2% in the semidark and dark zone, respectively (Figs. 1a and 2a,b). The semidark area was dominated by the EP (*Erylus mammillaris* and *Penares euastrum*) group (2.91 m^2^), followed by folious red algae (1.97 m^2^) and hydroid assemblages (1.78 m^2^), while the less represented taxa were the AI (*Aaptos aaptos* and *Iricinia variabilis*) group (0.013 m^2^), *Chondrosia reniformis* (0.031 m^2^) and the only homoscleromorphan sponge recorded, *Oscarella lobularis* (0.047 m^2^) (Table 2; Fig. 1a and 2a). *A. aerophoba* was instead the dominant component of the dark biocenosis (5.31 m^2^), together with EP group (4.01 m^2^) and hydroids (1.31 m^2^) (Table 2; Figs. 1a and 2a). Again *O. lobularis* and *C. reniformis* were among the taxa with the lowest coverage (0.005 and 0.024 m^2^, respectively), along with *Hymedesmia (Hymedesmia) pansa* (0.033 m^2^) (Table 2; Figs. 1a and 2a). Additionally, up to a total cover of 1.7 m^2^ (2.8%) of the cave was classified as “Not applicable” due to the low quality of orthomosaic caused by the particulate present in the water column (Table 2; Figs. 1a and 2a).

Overall, the sampling method using the Q50 (n = 40), covering an area of only 10 m^2^ (Table 1; Figs. 1b and S2), allowed to detect of all the 16 benthic categories, while the deployment of Q20 (n = 60, surveyed area = 2.4 m^2^) recorded a total of 15 categories, missing only *C. reniformis* (Tables 1 and 2; Figs. 1b and S2). Analysing each biocenosis separately, *C. reniformis* was not found in the dark area by the Q50, while the Q20 approach failed to detect *H. (H.) pansa, O. lobularis* and *Timea bifidostellata* in the dark area and the AI group in the semidark one (Table 2). However, the results obtained by each approach in terms of percentage cover do not seem to greatly differ (Fig. 2a–f), although some discrepancies can be observed: the CCA cover being underrated by both quadrat approaches in the semidark zone (Fig. 2c,e), or the hydroid assemblages of the dark biocoenosis being slightly underestimated by the Q20 (Fig. 2f) while overrated by the Q50 ones (Fig. 2d).

**Figure 3:**
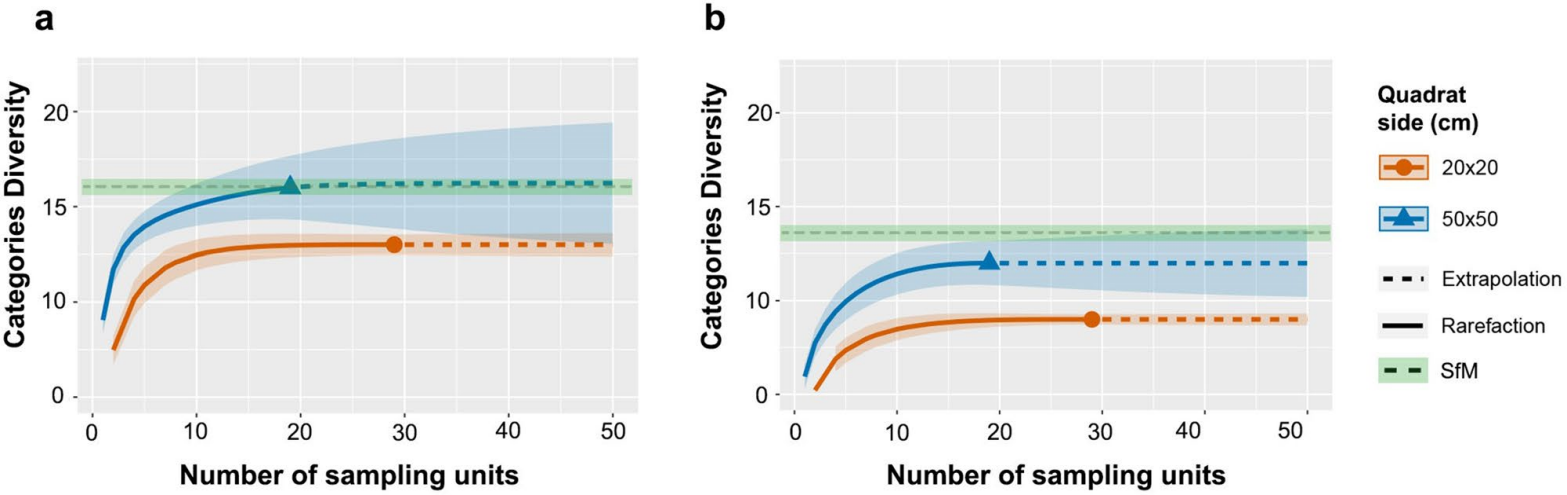
Sampling-unit-based rarefaction and extrapolation curves with 95% confidence intervals (shaded areas) for benthic diversity data considering the three applied methodologies in the (**a**) semidark and (**b**) dark areas of the cave. Q20 = 20 × 20 cm quadrats; Q50 = 50 × 50 cm quadrats; SfM = Structure from Motion photogrammetry.

In Fig. 3a,b the effect of the sampling effort was investigated better to explore the possible differences among the three approaches. In the semidark area, only the Q50 reached to identify all categories after 19 deployments, managing to detect 15 out of 16 benthic categories after deploying 10 sampling units (Fig. 3a). On the other hand, the Q20 identified 13 categories after 10 deployments, and still missed three at the maximum effort (n = 30) (Fig. 3a). In the case of dark biocoenosis, the applied sampling effort was not sufficient to reach the same number of categories as the SfM. Even so, the Q50 recorded 11 out of 13 categories after the deployment of 10 sampling units, while the Q20 only identified 7 for the same number of deployments (Fig. 3b).

### Assessment of *Lithophaga lithophaga* holes’ density and distribution

**Figure 4:**
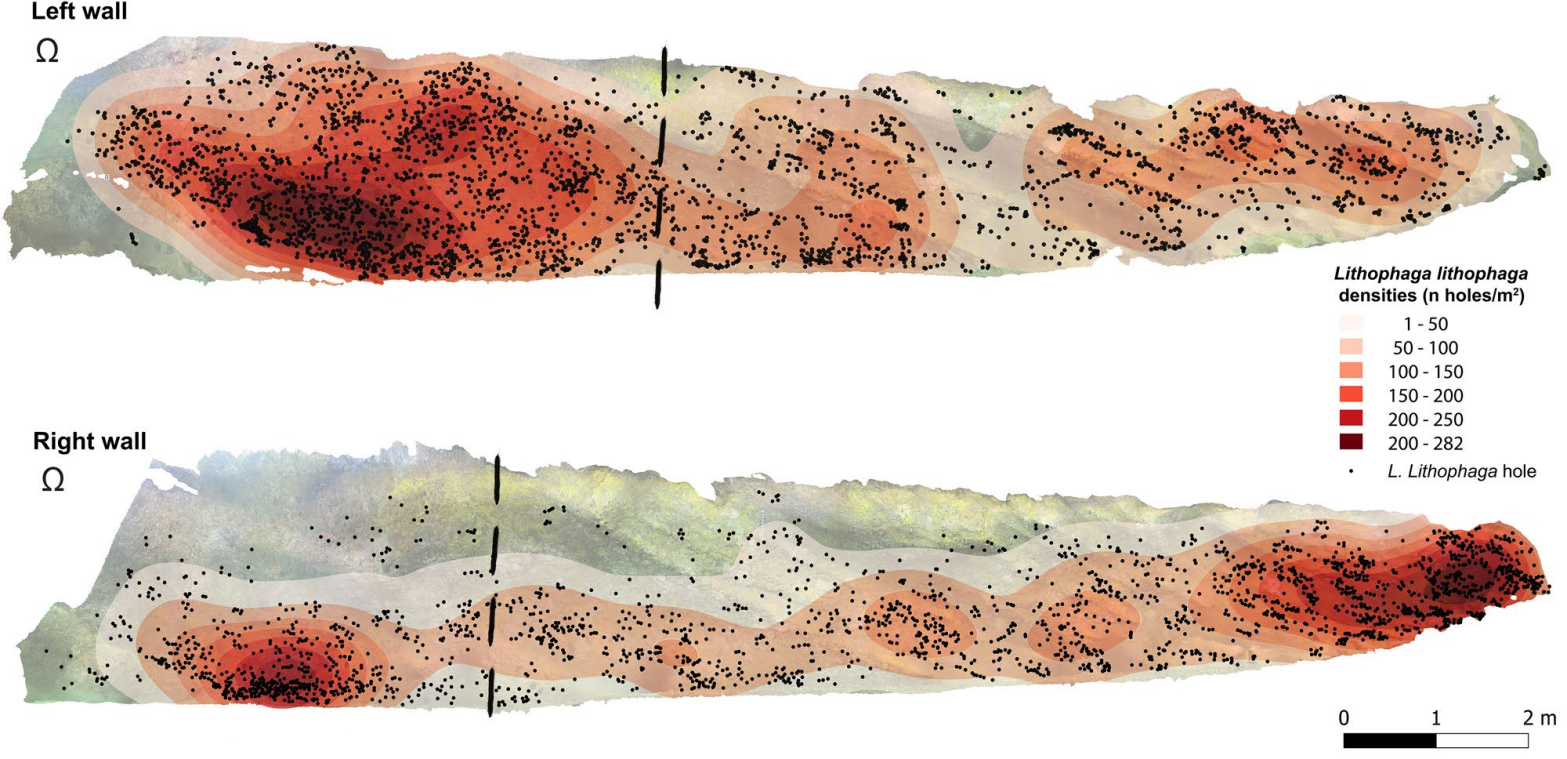
Distribution and density of date mussel (*Lithophaga lithophaga*) holes through the cave walls obtained by the analysis of Structure from Motion photogrammetry results. Dashed lines delimit the semidark and dark areas. The symbol Ω indicates the cave entrance. Created using QGIS software version 3.12 (http://www.QGIS.org).

Thanks to SfM-photogrammetry, *L. lithophaga* hole densities were recorded through the walls of the cave, obtaining an average density of 88.3 holes/m^2^ at cave scale (Fig. 4). Considering the two ecozones separately, a general decrease on the date mussel average densities was detected towards the inner parts of the cave, with a density of 102.95 holes/m^2^ in the semidark area against 78.65 holes/m^2^ in the dark one. Differences between the cave walls were also evident, being the semidark area of the left wall the ecozone holding the higher density (139.8 holes/m^2^) (Fig. 4). In addition, taking a close look, a preference of this species for boring into the vertical and sub-vertical parts of the walls may be observed (Figs. 4 and S3).

Both Q50 and Q20 methods overestimated *L. lithophaga* hole densities at cave scale (106.8 and 129.17 holes/m^2^, respectively). Considering the different areas separately, in the semidark zone the Q20 obtained very similar results to the SfM approach (105 holes/m^2^) whilst the Q50 method underestimated hole densities (83.7 holes/m^2^). Conversely, in the dark area it occurs just the opposite, obtaining a density value of 88.2 holes/m^2^ with the Q50 and 100.83 holes/m^2^ with the Q20.

**Figure 5:**
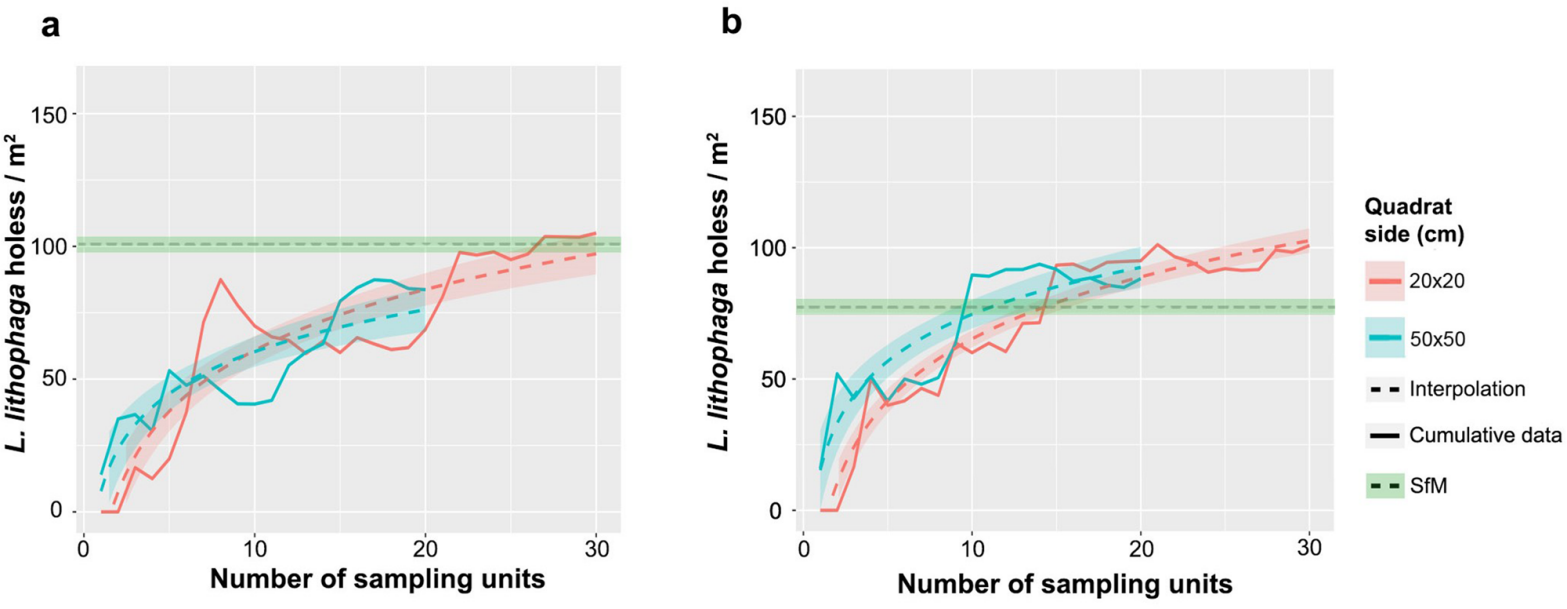
Interpolation curves for *Lithophaga lithophaga* holes’ density data in function of the sampling effort for (**a**) semidark and (**b**) dark area. Green line = results obtained by the Structure from Motion (SfM) approach analysis of the whole cave is presented as the green line. Q20 = 20 × 20 cm quadrats; Q50 = 50 × 50 cm quadrats.

The influence of sampling effort on the determination of *L. lithophaga* hole densities for the two considered quadrat sizes is presented in Fig. 5a,b. In the semidark area both quadrat approaches underestimated *Lithophaga* species with a relatively small number of sampling units, however the Q20 managed to obtain similar results that the SfM when reached the maximum sampling effort (Fig. 5a). On the other hand, in the dark area, with the deployment of 10 sampling units both quadrat approaches came quite close to the ground truth values, slightly overestimating hole’s density at its maximum sampling effort (Fig. 5b).

### Seascape metrics

**Figure 6:**
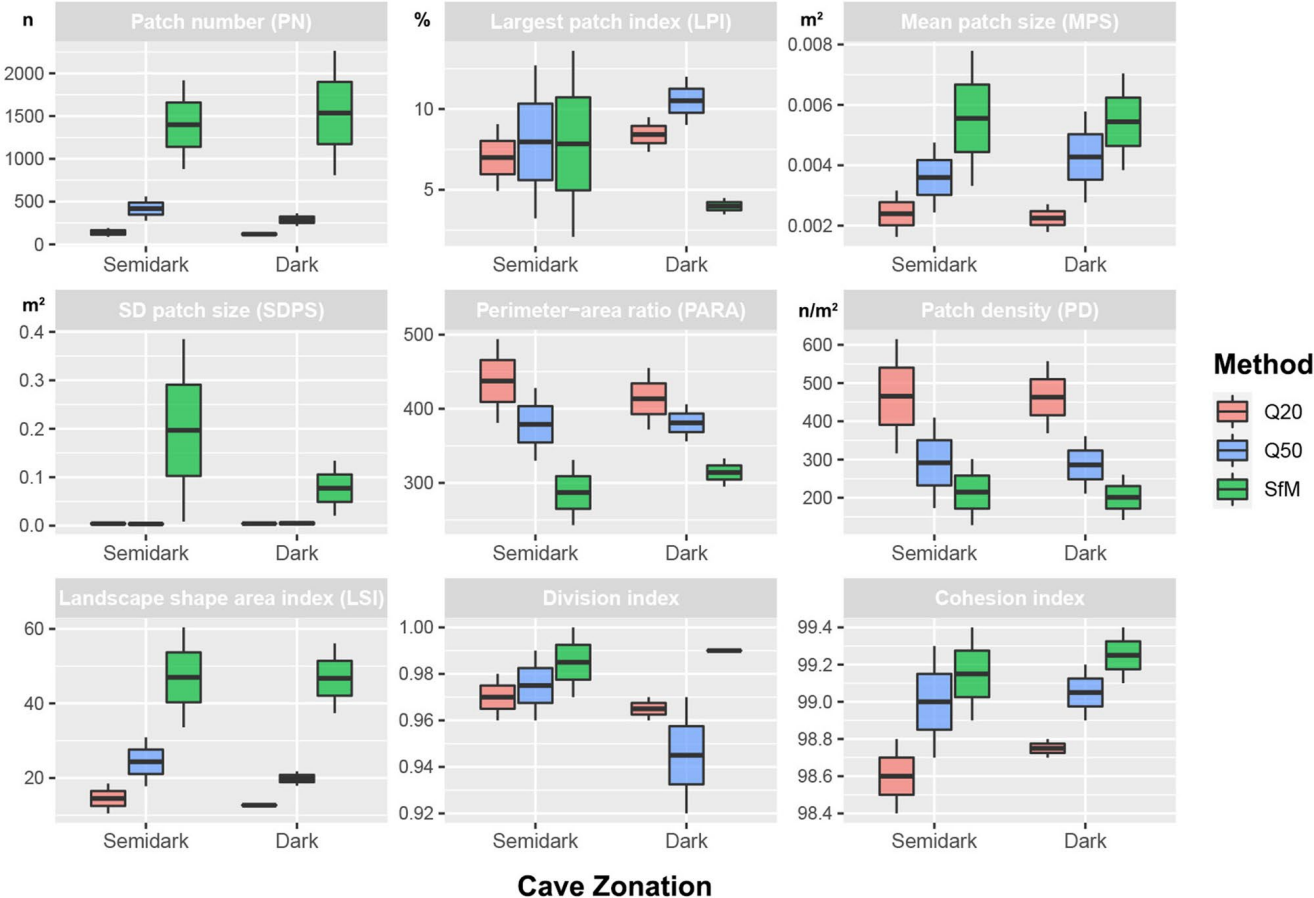
Seascape metric scores obtained for the three methodologies applied in the two cave zones. Q20 = 20 × 20 cm quadrats; Q50 = 50 × 50 cm quadrats; SfM = Structure from Motion photogrammetry.

Indices values at seascape level were calculated, according to Table 3, and presented as supplementary material in Table S1. Comparing the results obtained applying SfM, an increase in the abundance of PN and in the PARA index towards the inside of the cave was observed (Fig. 6). On the other hand, LPI and SDPS presented higher values in the semidark area, while MPS was comparable among the two zones (Fig. 6). Differences have also been recorded among sampling methodologies: (i) both quadrat approaches obtained lower values for PN, MPS and SDPS than SfM, (ii) quadrats recognized more significant patches (> LPI) in the dark biocoenosis, and, in terms of shape, (iii) SfM found more complex patch shapes (> PARA) in the dark area, Q20 in the semidark area, while Q50 registered similar values for both ecological zoned (Fig. 6).

**Table 3:** Seascape descriptors calculated at category and seascape levels for both ecological zones of Grotta Azzurra.

Index		Brief description
Patch number, size, and shape	Patch Number (PN) (n patch)	Number of patches. It describes the fragmentation of a category, however, does not necessarily contain information about the configuration or composition of the category
	Largest Patch Index (LPI) (%)	Correspond to the percentage of the seascape interested at the single largest patch; where 100 would mean that the seascape consists in a single large patch and approach 0 as this dominant patch decreases in size
	Mean of Patch Size (MPS) (m^2^)	The metric summarises each category as the mean of all patch areas belonging to category i
	Standard Deviation of Patch Size (SDPS) (m^2^)	The metric summarises each category as the standard deviation of all patch areas belonging to category i
	Mean Patch Area Perimeter Ratio (PARA)	It summarises each category as the mean of each patch belonging to category i. The perimeter-area ratio describes the patch complexity in a straightforward way. As PARA increases, patches become more complex
Dispersion/Aggregation	Patch Density (PD) (n patch / 1 m^2^)	Number of patches per area of the landscape, please notice that it is standardized to area in order to be comparable among landscapes with different areas
	Landscape Shape Area Index (LSI) (%)	This index measures the perimeter-to-area ratio for the whole. It ranges from 1 to infinite; where 1 would mean that the seascape consists in a single patch and increases as the patches become more disaggregated
	Division Index (DIVISION) (ratio)	Correspond to the probability that two pixels chosen randomly in the seascape are not located inside the same patch; it presents higher values when a seascape is highly segmented in separate patches
	Cohesion Index (COHESION) (%)	It characterises the connectedness of patches belonging to a category. It assesses if patches of the same category are located aggregated or rather isolated and thereby gives information about the landscape configuration
Diversity	Shannon’s Diversity Index (SHDI)	This index is based on the information theory (Shannon & Weaver, 1949) and its value represents the amount of “information” per category. Takes both into account the number of categories and the abundance of each category, thus a high value corresponds to a higher number of category types and/or evenness
	Simpson’s Diversity Index (SIDI)	The value of Simpson’s index reflects the probability that two random patches belong to the same category. It is less sensitive to rare categories than SHDI. The higher the value, the greater the diversity
	Shannon’s Evenness Index (SHEI)	This index is based on the proportion of max. SHDI on the distribution of area among patch types; its value is 1 when the area covered by the different patch types is evenly distributed. It is understood as a measure of dominance
	Simpson’s Evenness Index (SIEI)	This index is based on the ratio between the actual SIDI and the theoretical max. SIDI. Its value is 1 when the area covered by the different patch types is evenly distributed

All metrics not presenting units in the table are dimensionless.

**Figure 7:**
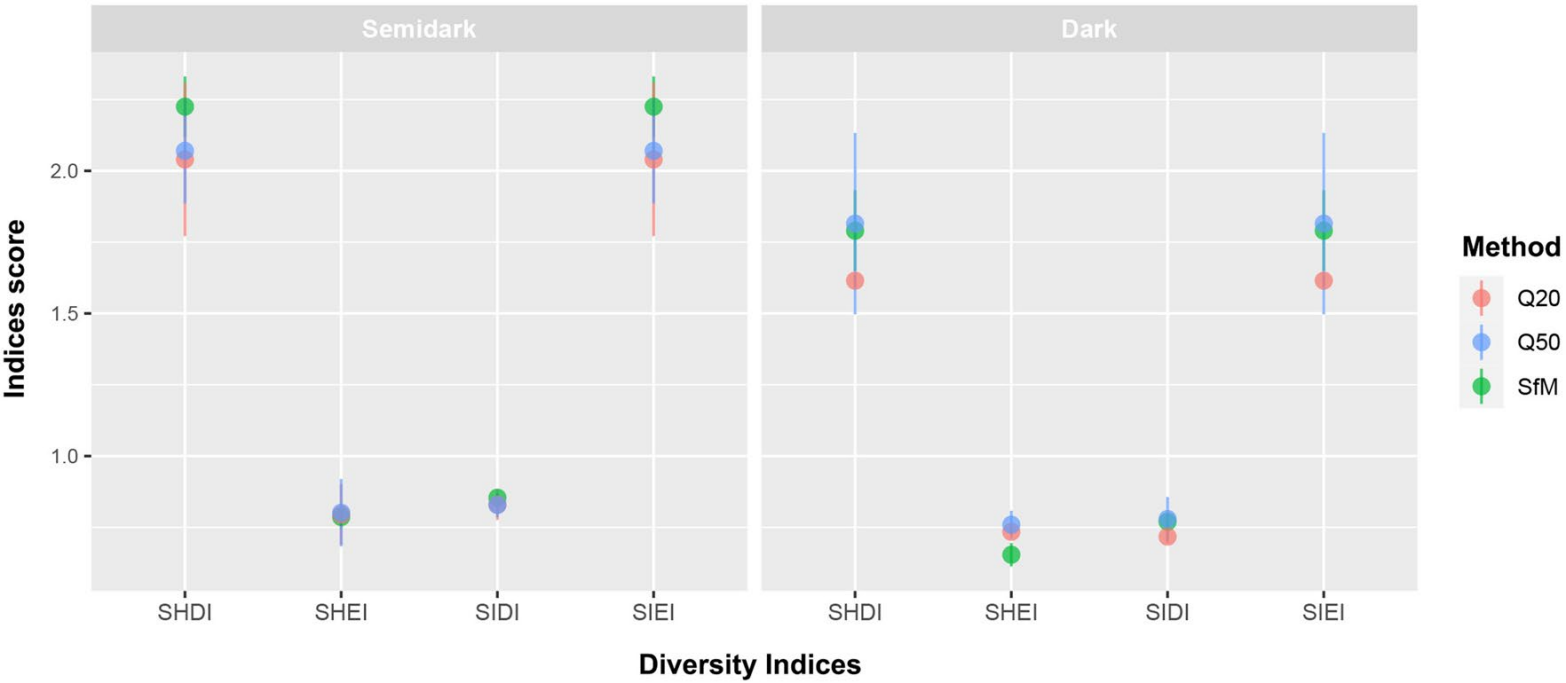
Diversity Indices scores obtained for the three different methodologies inspected in the two cave zones. SHDI = Shannon Diversity Index; SIDI = Simpson Diversity Index; SHEI = Shannon Evenness Index; SIEI = Simpson Evenness Index; Q20 = 20 × 20 cm quadrats; Q50 = 50 × 50 cm quadrats; SfM = Structure from Motion photogrammetry.

Regarding aggregation indices, no great discrepancies were found between the two cave zones by any of the methods (Fig. 6), yet Q20 and Q50 overestimated the average patch density present in the cave (Fig. 6). Finally, the results obtained with SfM for diversity and evenness found higher SHDI and SIEI values in the semidark area, indicating a more diverse and equally distributed community than the dark area (Fig. 7). Additionally, even though Q20 slightly underestimated both SHDI and SIEI, the values obtained with the three methods are similar (Fig. 7).

Seascape indices were also calculated at category level, with all three approaches obtaining comparable results (see Table S2). Regarding patch number size and shape indices, for PN and PD, *Clathria (Microciona) armata* and *H. (H.) michelei* were identified as the more abundant and dense species in the semidark area, while in the dark one they were tunicates and *A. aerophoba*; on the other hand, *C. reniformis*, *O. lobularis* and AI group hold the lower values in both biocoenosis (Table S2). Red folious algae and hydroid assemblages were the categories presenting the higher LPI, MPS and SDPS in the semidark area, while EP group, *A. aerophoba* and again hydroids where the highest in the dark zone. The categories with the smallest patch, average size and size variability were *C. (M.) armata*, EP group, *H. (H.) pansa*, *O. lobularis*, *Rhabderemia topsenti* and tunicates in both biocenosis (Table S2).

Looking at aggregation indices, LSI, division, and cohesion gave us an idea of the configuration and connectedness of the patches. *H. (H.) michelei*, *C. (M.) armata* and *O. lubularis* resulted in the categories more disaggregated and less connected in the semidark areas, while AI group, hydroids and red folious algae were the categories holding the most compact and aggregated distributions. Towards the dark area, hydroids, and *H. (H.) michelei* were the more aggregated categories against tunicates, *O. lobularis* and *A. aerophoba* which were highly disaggregated and less compact (Table S2). The PARA index was the only exception in the observed trend. In fact, the three methods considered showed highly different results: SfM recognized *O. lobularis, H. (H.) pansa* and *Tedania anhelans* as the species with the most complex patches shapes (> PARA), while both quadrat approaches identified *C. reniformis*, tunicates, *C. (M.) armata* and *O. lobularis* (Table S2).

## Discussion

All taxa identified in Grotta Azzurra are well-known and typical of Mediterranean marine caves^9, 31, 61^, and their abundance and distribution along the two cave zones agree with the previous literature^3, 4, 6, 62^. Porifera was the most representative group, defining Grotta Azzurra as a “sponge realm”, as previously observed for other caves^37, 60^. Overall, the most abundant sponges in terms of coverage and number of patches along the entire cave (*Aplysina aerophoba* and the EP group, formed by *Erylus mammillaris* and *Penares euastrum*) are also described in the literature among the most common species and genera of Mediterranean marine caves^31, 61, 63^. Additionally, *A. aerophoba* specimens presented an unusual growth habit characterised by “cushion-shaped individuals connected by abundant thin branching processes and forming large encrusting plates'' (Fig. S4), a type of growth only recently described by Costa et al.^63, 64^ in four semi-submerged Italian caves.

Hydroids were another abundant taxon in both biocenosis, and the only class of Cnidaria found in Grotta Azzurra. Cnidarian species (e.g., *Leptopsammia pruvoti* Lacaze-Duthiers, 1897, *Parazoanthus axinellae* (Schmidt, 1862)*, Astroides calycularis* (Pallas, 1766), among others) are typical components of cave communities, especially on the ceilings and walls of the entrance and semi dark zones. However, their presence is highly related to various abiotic factors, such as depth, water movement and sedimentation^5, 65^. The peculiar environmental constraints of this semi-submerged cave (i.e., tunnel-shaped morphology, high hydrodynamic conditions) strictly select the species that can exploit this challenging niche. Endolithic, encrusting and soft-bodied species can face the high-water turbulence, dominating the benthic assemblage^66, 67^. These features allowed hydroids to proliferate and form significant patches along the walls, and their presence also in the deeper parts of the cave points out that high rates of water movement are not limited to the cave entrance^61, 68, 69^ thanks to the waves’ reflection inside the cave. Additionally, hydroid patches presented a peculiar and marked distribution, occupying almost exclusively the overhangs of the walls’ roughness, a pattern possibly driven to intercept the maximum intensity of water movement^68, 69^.

In terms of species richness, percentage cover of identified taxa and most of the seascape metrics at category level, the three sampling methodologies here applied (SfM, Q50 and Q20) obtained comparable results, highlighting the reliability of traditional approaches in the general characterization of a cave community, and suggesting the comparability of data obtained by traditional and more innovative methods. Nevertheless, it must be considered that the sampling effort implemented in this study for Q50 and Q20 was particularly high compared to the typical sampling effort applied in caves^35, 70^, usually related to bottom time and other logistic constraints.

The results of random quadrat deployments can strongly vary depending on the quadrat size and sampling effort, limiting its potential to capture uncommon species distributions (Table 4)^35, 36^. The Q50 seemed to be a more cost-effective solution than Q20, obtaining more similar results to SfM. Even so, a limitation on its implementation in these habitats should be acknowledged; in fact, caves often present narrow passages where carrying a frame of such dimensions could not be possible or its manipulation risks to damage organisms protruding from the surface, especially species with fragile skeletons (Table 4)^71^. Additionally, in terms of taxa density estimations, both quadrats’ approaches overestimated their values at cave and category levels (Table 4). A similar situation occurred for the estimation of *Lithophaga lithophaga* hole densities. Conversely, the application of SfM-photogrammetry allowed not only to define density values accurately, but also to record their distribution along the cave walls. The density patterns of *L. lithophaga* holes could possibly be explained by the hydrodynamics occurring in the cave: in fact, by comparing its distribution with the structural complexity of the walls, it is clear how the morphology and orientation of the substrate play a crucial role on the settlement of this species, which usually thrives in sub-vertical to vertical carbonate substrates, thus avoiding high sedimentation rates^72, 73^. Due to the high price and demand of the date mussel in the Mediterranean Sea, this species is still intensively collected despite the laws and marine policies forbidding it. For its extraction, the rock is heavily damaged, causing a dramatic simplification of the biotic and structural composition of the substrate, leading to an impoverishment of the entire community^74, 75^, a phenomenon widely spread in the Conero Riviera (Fig. S1).

**Table 4:** List of advantages and disadvantages of the compared methodologies inspected in this study.

Protocol	Estimated time consumption (h)		Advantages	Disadvantages
	Sampling	Analysis		
SfM	1	100	Establish a complete baseline Capture 3D structural complexity Multiscale approach Science outreach application	Require longer bottom times Require longer analysis times Depending on the extension and scale, possible need of a powerful workstation
Q20	0.5	15	Shorter bottom times No need of a powerful workstation	May miss uncommon species Densities overestimation
Q50	0.3	30	Shorter bottom times No need of a powerful workstation	May miss uncommon species Densities overestimation Size of the frame

Q20 = 20 × 20 cm quadrats; Q50 = 50 × 50 cm quadrats; SfM = Structure from Motion photogrammetry.

In this context, when applicable, SfM photogrammetry should be considered as an additional and complementary tool for marine cave monitoring. Applied over time, short, medium, and long-term changes could be recorded from seascape to individual level, down to a mm scale^48, 76^. By the 3D reconstruction of the substrate morphology, a more complete picture of the biological distribution patterns and the processes driving them can be provided^47, 77^. The implementation of systematic monitoring plans including photogrammetry would also help lawmakers in the creation of ad-hoc management and protection measures targeting specific threats affecting marine caves locally^46^. Furthermore, the obtained 3D models represent a powerful ally for public outreach activities, raising awareness by developing interactive experiences where people can explore these hidden habitats (e.g., Fig. S5)^78, 79^.

Nonetheless, given the intrinsic risks related to cave diving^34^, some considerations must be made before implementing the technique in underwater caves, especially in terms of cave’s depth, size and morphology to ensure SfM applicability. Moreover, depending on the main objective of the study, the desired spatial and taxonomical resolution of the digital outputs, and the extension of the area to be mapped, the sampling, processing and annotation times may increase exponentially (Table 4). Thus, a cost-benefits evaluation must be made to evaluate the different techniques available, reducing operators’ risks and maintaining a cost-effective monitoring effort^9, 31^. In the assessment of large areas, a more powerful working station or cloud-based processing solutions may be needed to process the imagery dataset^47^. Additionally automated and semi-automated tools must be considered to support and speed up manual annotation process, improving the efficiency of the method^80–84^.

Although the rise of compact waterproof cameras provides a high-quality, reasonably-price solution, which suggest photogrammetry as an affordable and attractive method for both recreational and scientific scuba divers^57^, a proper training and the implementation of a standardized protocol is essential for a good application of the technique and the acquisition of accurate reconstructions^49, 56^.

In conclusion, even if quadrats work great detecting the main coverages and trends in homogeneously distributed benthic communities, when it comes to sparse heterogeneous communities (e.g., dark cave biocoenosis) or rare organisms, they may overlook some of its components. Underwater photogrammetry demonstrated to be a suitable non-invasive technique to record the cave benthic assemblages, besides the calculation of a group of carefully selected seascape indices proved to be a comprehensive way to describe the present spatial pattern. A first baseline was established for Grotta Azzurra: the definition of the current distribution of its biotic component represents a crucial information in these times of rapid community shifts due to the synergy of climate change and human stressors^10, 29^. A bright future awaits photogrammetry in the field of marine environments monitoring thanks to its huge potential and versatility, together with the exponential progress of computer vision, robotics, and machine learning^80–84^.

## Materials and methods

### Study site

**Figure 8:**
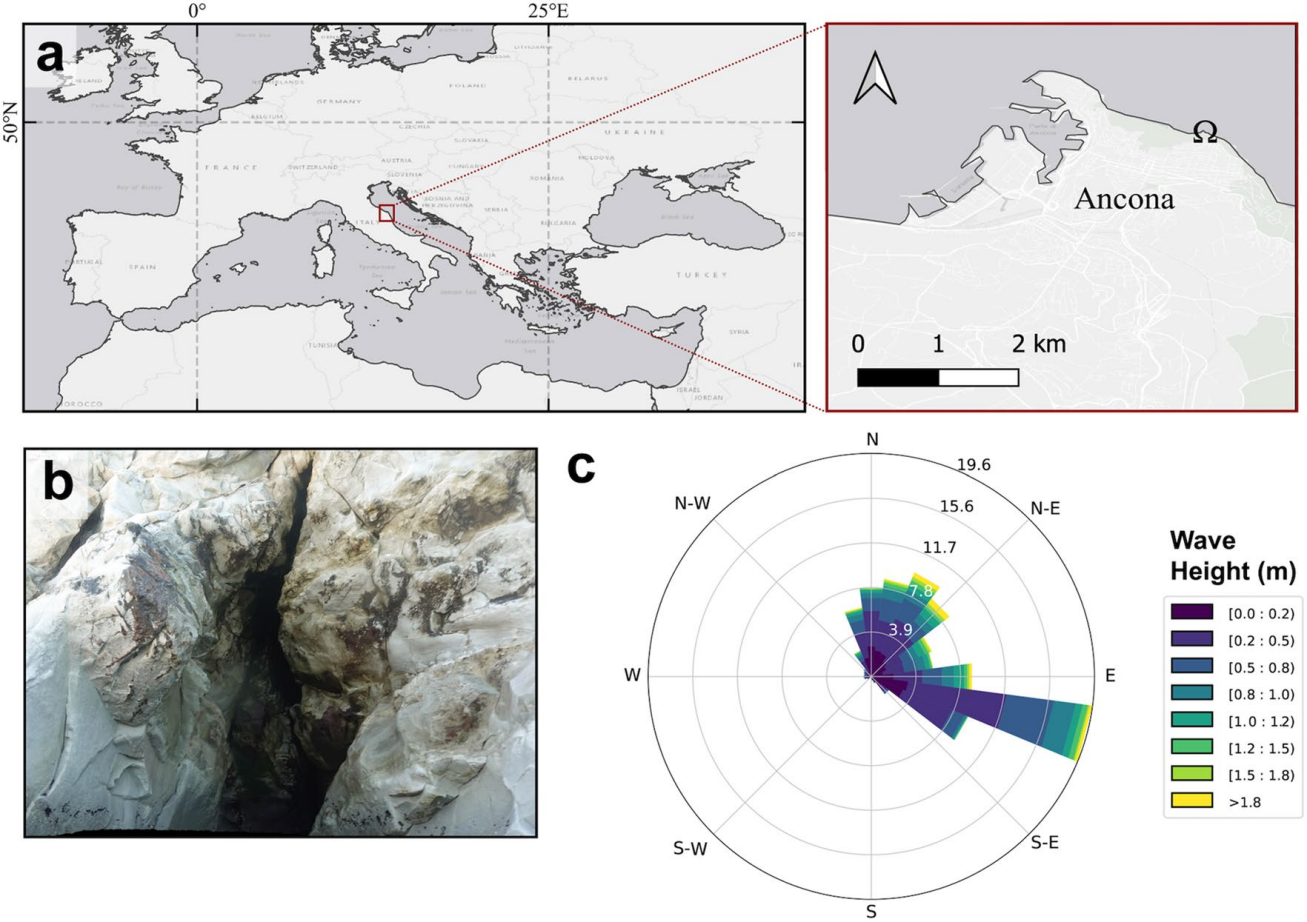
(**a**) Location map of Grotta Azzurra (Ancona, Italy), marked with the symbol Ω. (**b**) Detail of the emerged part of Grotta Azzurra entrance. (c) Wave rose of significant wave height, corresponding to the 2021 full year period at the near-shore waters in front of the cave entrance. Created using QGIS software version 3.12 (http://www.QGIS.org).

The Conero Riviera (Ancona, Italy) is a shoreline that suffered from strong artificialization over the last century, with some of its natural substrates modified or replaced by cemented structures^85^. A series of shallow marine semi-submerged caves are present along its coastal cliffs^86^, and in this study the bigger of these caves was explored, locally known as Grotta Azzurra (43° 37′ 17.2" N, 13° 31′ 38.4" E) (Fig. 8a). Its opening represents a big fissure facing north into the calcareous walls, starting a few metres above the sea surface (Fig. 8b), and continuing down to 4.5 m depth. With 15 m in length, this semi-submerged tunnel-shaped cave reaches its maximum depth at its entrance (4.5 m), presenting a floor mainly composed of gravels and small rock aggregations. The dominant wave direction in the area is E–SE (Fig. 8c), leaving the cave partially sheltered from the direct action of waves. Nevertheless, the lack of thin sediment accumulation inside the Grotta Azzurra, despite the high sediment charge that characterises the bottoms of the Conero Riviera^85^, suggests a moderate to high hydrodynamic regime.

### Data acquisition and photogrammetric processing

The sampling strategy for the characterization of the sessile benthic community present inside Grotta Azzurra consisted of the coupling of two sampling methodologies, each one implemented during a dedicated dive. In the first place, a photographic sampling of the cave was performed during early September 2021. A GoPro HERO8 Black (Woodman Labs, Inc., San Mateo, CA, USA) equipped with an artificial lighting system composed of two AKKIN 5000 underwater lights was used. Along the walls and the floor of the cave, a series of metric references were placed to scale-up and control the accuracy of the digital reconstructions. The camera was set to time-lapse mode at 2 frames per second, and the diving operator adapted the sampling path to capture the whole cave topography by carrying out a vertical boustrophedonic pattern, ensuring a minimum of 60% overlapping among consecutive images^45, 47^. Maintaining an average distance from the substrate of around 40 cm, the images were homogeneously illuminated. A total of 3,600 images were collected and controlled to ensure picture quality before being imported into Metashape v. 1.8.2. (Agisoft LLC, St. Petersburg, Russia). Images alignment was performed using high accuracy generic pair selection settings to produce the point clouds, limiting the key points identification to 100,000 and the tie point limit to 10,000 common feature points. Meshes were produced by the arbitrary three-dimensional surface type from the depth maps data, medium face count and interpolation disabled. To scale up the model, 5 metric references were manually detected in the imagery dataset and used to create scale bars in the reference settings. Finally, an orthomosaic of each wall was produced by mosaic blending mode from the mesh surface data and exported as tiled tiff in a local coordinate system (m). The overall photogrammetric process to generate both the digital reconstruction of the cave and the couple of orthoimages took 16 h of processing time using a Lenovo Legion laptop (Beijin, China) with an Intel Core i7-9750H 2.60-GHz processor (Intel Corporation, Santa Clara, CA, USA), 32 Gb RAM and a graphic card NVIDIA GeForce RTX 2060 (NVIDIA Corporation, Santa Clara, CA, USA).

After identifying the organisms occurring in the orthomosaic, cover percentages of the various sessile organisms were calculated to assess the abundance and the distribution patterns of benthic species in the two ecological zones, semidark and dark, following the cave zonation defined by Pérès and Picard^3^. When the sponges were not identifiable at species level, the individual's location in the digitised cave walls was noted using the orthomosaics. To increase the taxonomic resolution, a second dive was performed to collect a fragment as small as possible of each unidentified sponge. The collected samples were then fixed in ethanol 95% and processed as described by Rützler^87^ for the preparation of slides. Specimens were then identified at species level using a Nikon Eclipse Ni compound microscope. Slides were stored as a reference and deposited at the Zoology Laboratory Collection (Department of Life and Environmental Sciences), Polytechnic University of Marche (Ancona, Italy). Taxonomic identifications were carried out based on Systema Porifera^88^, “Proposal for a revised classification of the Demospongiae (Porifera)”^89^ and the World Porifera Database^90^.

In this study, no live vertebrates and/or higher invertebrates have been used for experimental purposes.

### Digitalization and single patch analyses

The orthomosaics produced for both cave walls were imported into QGIS software v. 3.12^91^, the different mega-epibenthic sessile taxa were manually digitised into scaled polygons or patches and classified at the lowest taxonomic level possible. Nonetheless, in some cases, it was not possible to differentiate between some of the identified species, therefore they were clustered into a group of species (Table 2). In particular, the AI group included the sponge species *Aaptos aaptos* (Schmidt, 1864) and *Ircinia variabilis* (Schmidt, 1862), and the EP group consisted in the aggregation of the sponges *Erylus mammillaris* (Schmidt, 1862) and *Penares euastrum* (Schmidt, 1868) (Table 2). Serpulids and vagile species were not considered for the analysis. The whole digitization process required 10 days (i.e., 70 h). Both walls were fully segmented and classified, including the bare substrate, and each segmented category’s surface area (m^2^) was calculated. *Lithophaga lithophaga* occurrence was accounted for and digitised throughout the entire walls. The fact that among all holes inspected, only *L. lithophaga* individuals, or shell fragments, were found inside the holes, made us assume that the date mussel was only responsible for all the holes present in the cave. However, to maintain the non-invasive character of the approach and to contain bottom time during sampling activities, it was not possible to confirm if each of the recorded holes was currently inhabited by the bivalve. *L. lithophaga* density was calculated in each cave zone by dividing the number of holes with the surface of each zone and expressed as *L. lithophaga* holes/m^2^. Additionally, a heatmap (Fig. 4) was produced in QGIS by using the heat map render in the layer styling panel with a 1 m radius to visualize density changes through the cave walls.

Two ecological zones were considered for the analysis, the semidark (i.e., entrance of the cave, with sciophilous macroalgae) and dark (i.e., inner section of the cave) biocoenoses, applying the model of cave zonation defined by Pérès and Picard^3^. To compare the results obtained by photogrammetry and to explore the effects of different sampling efforts, a second approach was also applied implementing a virtual simulated quadrat deployment in QGIS. Two quadrats’ sizes were considered, 20 × 20 cm (Q20) and 50 × 50 cm (Q50), with 60 and 40 deployments respectively (Table 1). To this end, the tool “Random Points Inside Polygon'' was applied to place the quadrats’ centroids over the walls, discarding those ending up partially out of the orthomosaics or overlapping^45^. Secondly, the already classified vectorial layers were clipped by each digital quadrat to extract species’ surface covers and the *L. lithophaga* holes densities.

### Seascape metric estimations

Key seascape metrics were calculated using *landscapemetrics* R package^92^, a drop-in replacement for FRAGSTATS software^93^. Using a rasterized version of our digitised cave community as an input, this package allowed the quantification of seascape characteristics at a multispatial level by considering patches size, shape, and distribution. A series of seascape descriptors (Table 3) were selected based on the literature and its ecological relevance^45, 46, 82, 83,94,95^, and estimated at both category and seascape levels for each ecological zone and sampling approach. For the analysis, the category “bare substrate” was not considered, to focus our spatial analytical efforts on the biotic components of the cave.

## Supplementary Information

Supplementary Information.

Supplementary Table S1.

Supplementary Table S2.

Supplementary Video S1.

## Data Availability

The datasets analysed during the current study are available from the corresponding author upon reasonable request.
