# Supplementary material for: Photogrammetry as a promising tool to unveil marine caves’ benthic assemblages
Source: Sci Rep. 2023 May 10;13:7587. doi: 10.1038/s41598-023-34706-7 (PMC10172382; doi:10.1038/s41598-023-34706-7)
Supplement: Supplementary file 1 — Supplementary Information. [file 41598_2023_34706_MOESM1_ESM.docx]

**Photogrammetry as a promising tool to unveil marine caves’ benthic assemblages**

Torcuato Pulido Mantas, Camilla Roveta, Barbara Calcinai, Cristina Gioia di Camillo, Martina Coppari, Veronica Marchesi, Teo Marrocco, Stefania Puce, Carlo Cerrano

Corresponding author: CAMILLA ROVETA

Dipartimento di Scienze della Vita e dell’Ambiente, Università Politecnica delle Marche, Via Brecce Bianche, 60131, Ancona, Italy.

[c.roveta@staff.univpmit](mailto:c.roveta@staff.univpmit); +390712204649

**Supplementary Information**


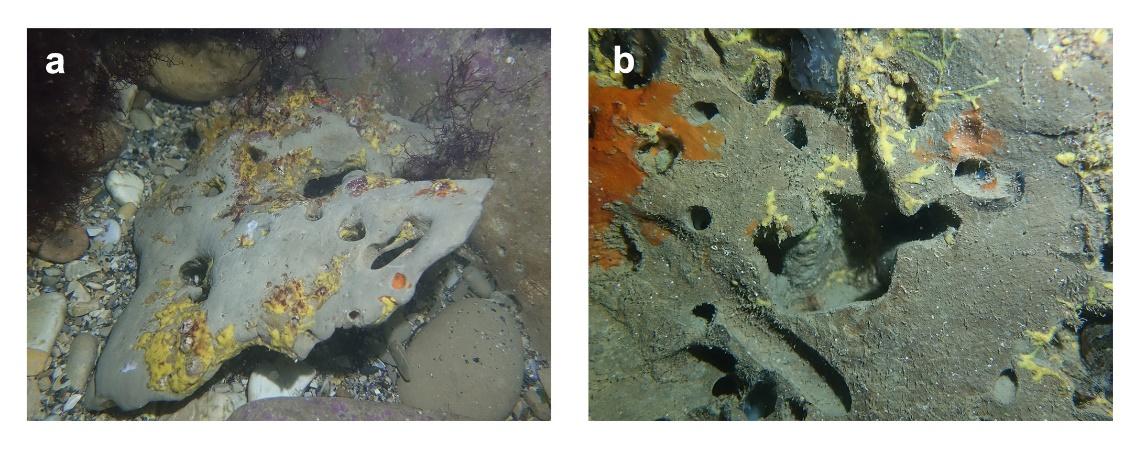


**Figure S1 –** Evidence of *Lithophaga lithophaga* illegal fishing at Grotta Azzurra marine cave (Ancona, Italy). (a) A piece of the cave wall found on the ground at the entrance of the cave; (b) the impacted wall presenting the empty *L. lithophaga* holes collected.

**Video S1 –** Virtual tour of the cave digital reconstruction.


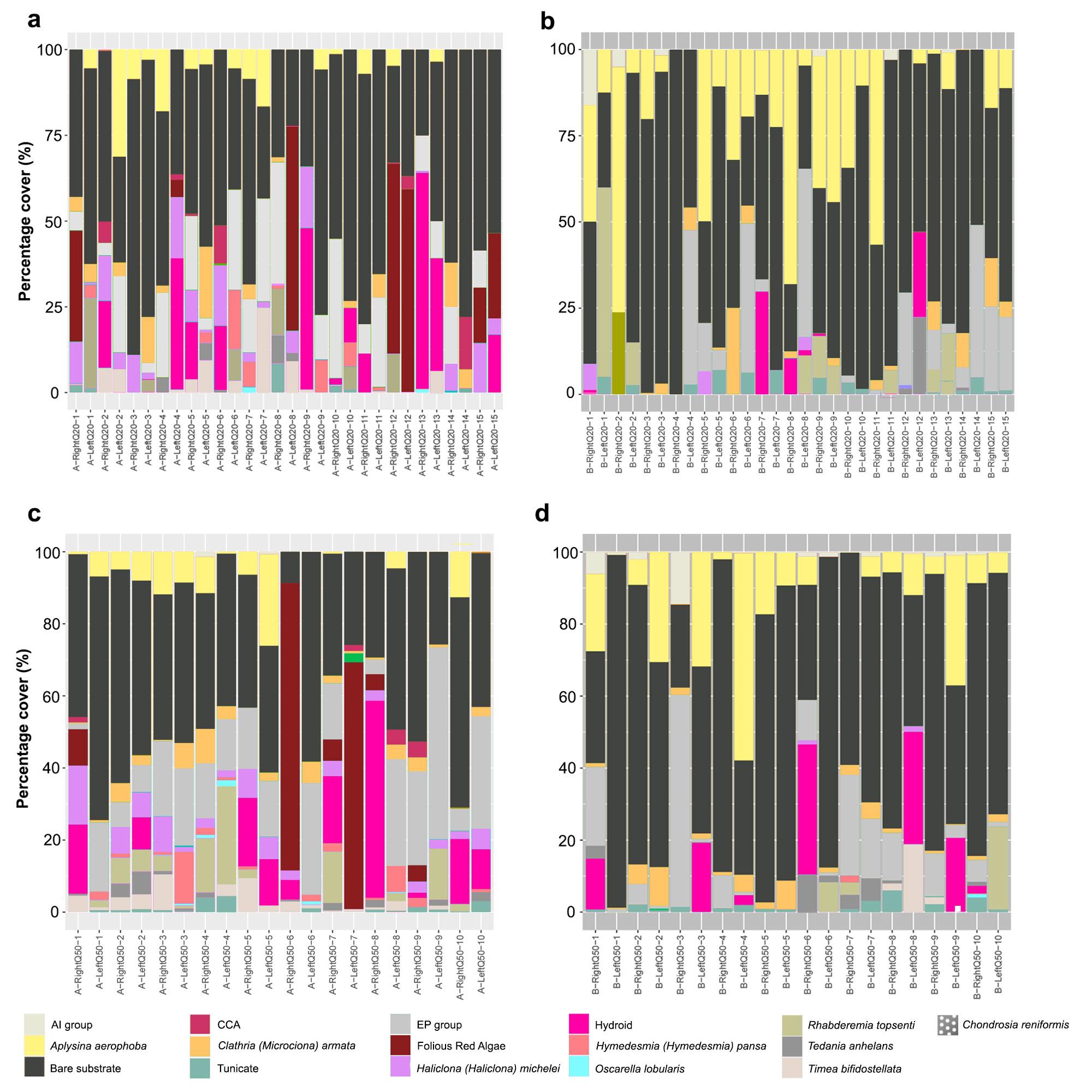


**Figure S2 –** Stacked bar plots representing the percentage cover of the benthic categories identified and of the bare substrate per: 20 x 20 cm quadrat approach in the (a) semidark and (b) dark zones; and 50 x 50 cm quadrat approach in the (c) semidark and (d) dark zones. For categories’ acronyms see Table1.


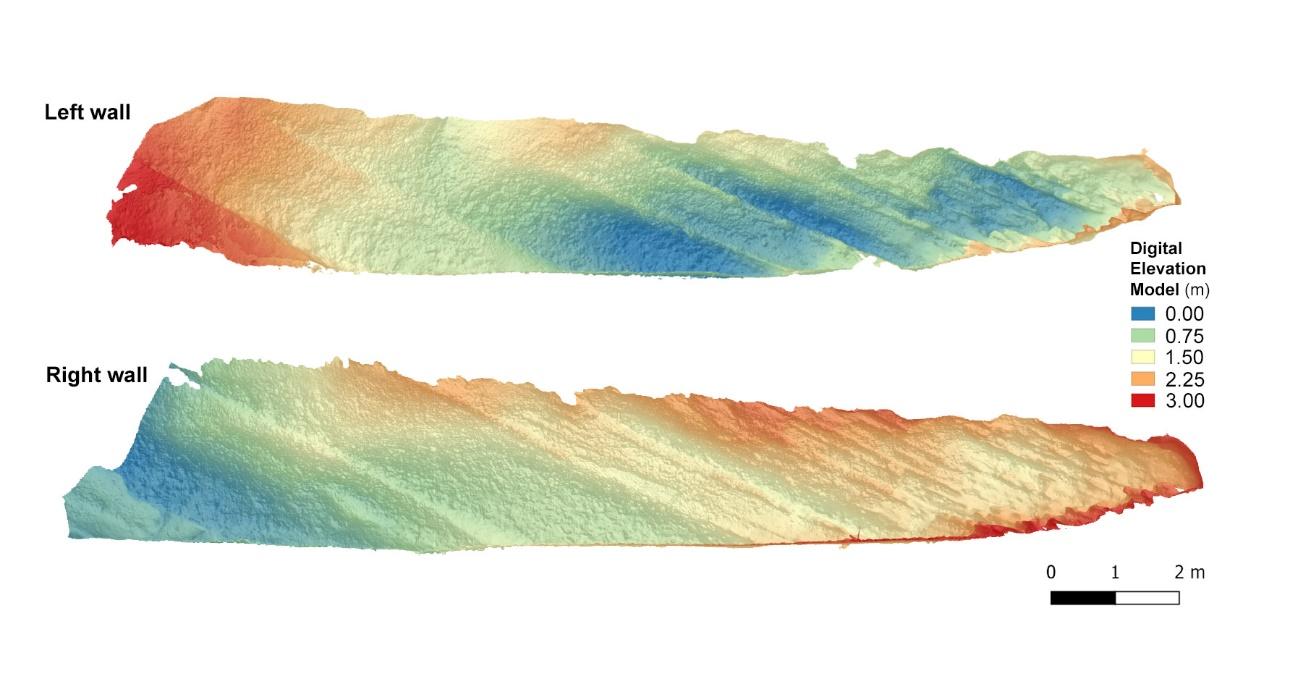


**Figure S3 –** Representation of the structural complexity of the cave walls by calculating the digital elevation model, created using QGIS software version 3.12 (http://www.QGIS.org).


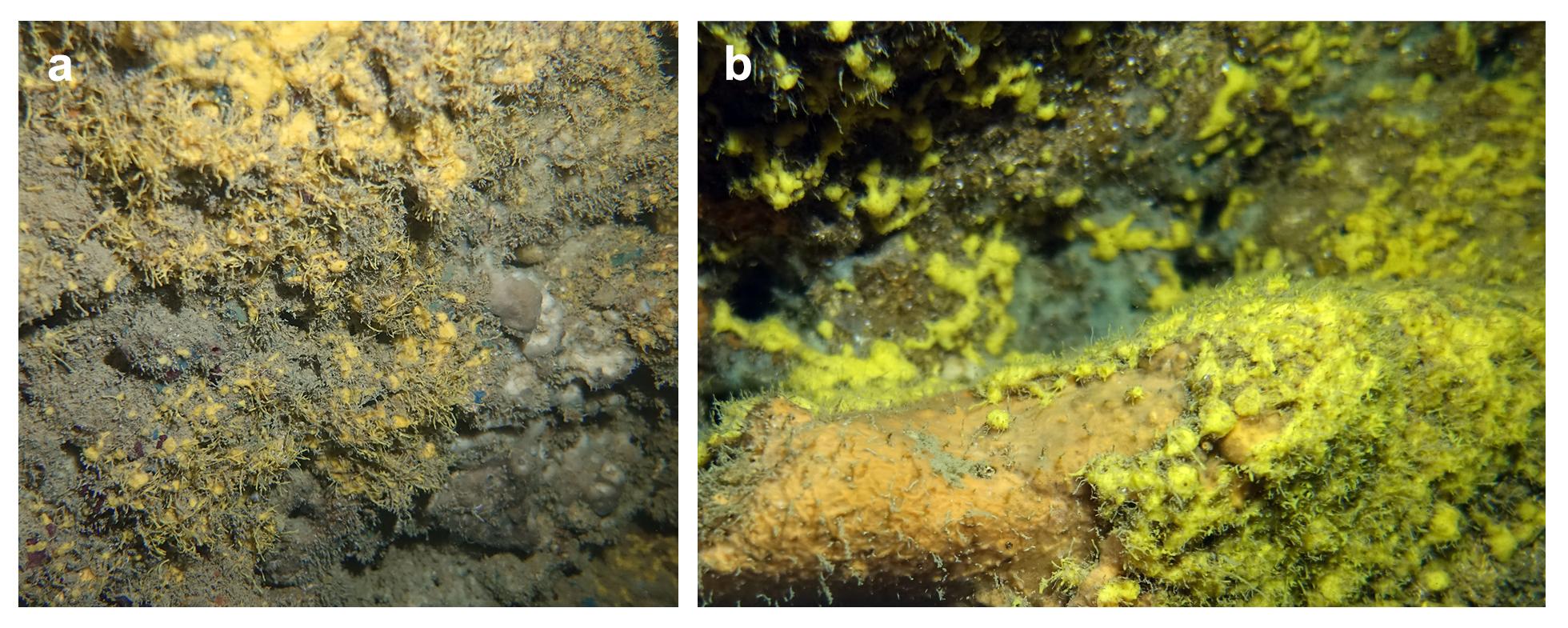


**Figure S4 –** Example of *Aplysina aerophoba* specimens, presenting the growth habit recently described by Costa et al. (2018, 2020).


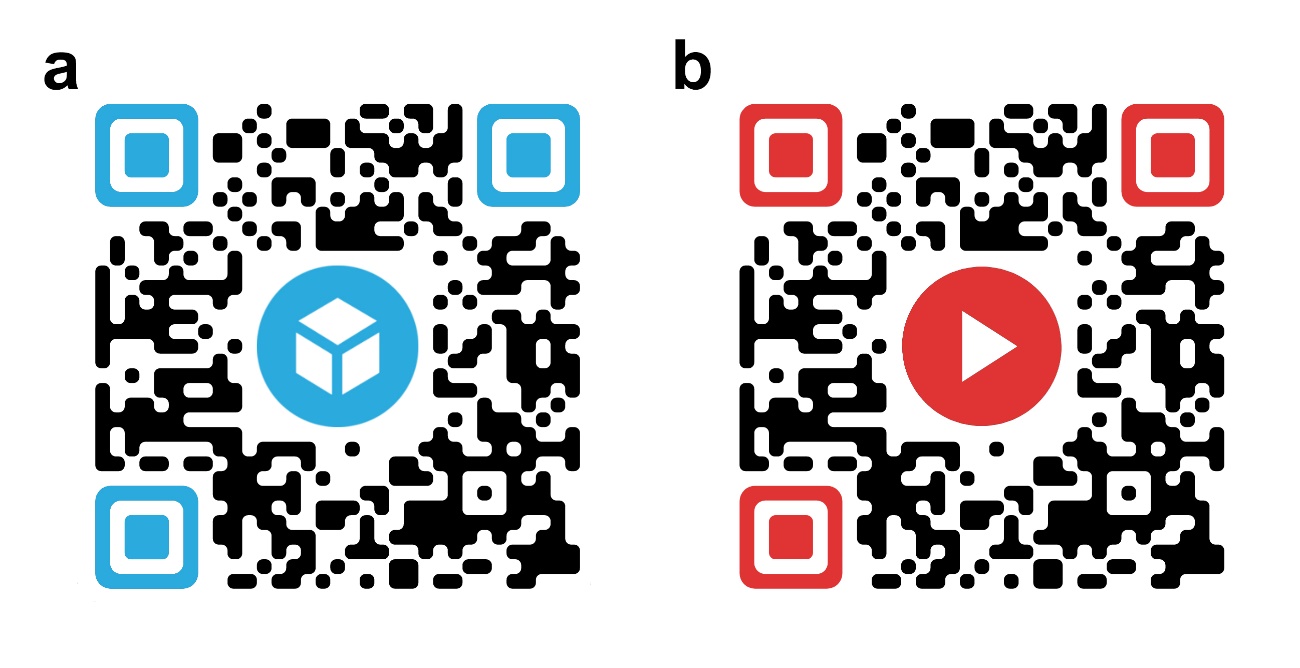


**Figure S5 –** Quick response (QR) codes: (a) to visit the 3D reconstruction of Grotta Azzurra marine cave in Sketchfab repository and (b) to have a virtual tour to the cave reconstructions hosted in YouTube.
